# Letter from Birmingham

**Published:** 1890-09

**Authors:** L. G. Hardman

**Affiliations:** Birmingham, England


### Correspondence.

---

BIRMINGHAM, ENGLAND, July 31, 1890.

MR. EDITOR—I had the pleasure of being present at the British Medical Association, which convened in this city on the 29th inst., and I give you some of the principal things of interest, thinking it will be of interest to your readers.

This great representative body of the medical profession of the British Isles met in the Maddin Institute.

The president elect, Mr. Wade, delivered his address at 8:30 o'clock in the evening. Subject, "Medical Education," in which he recommended six years' study, instead of the five which is required in this country now, before receiving any degree whatever.

He advocates, if the student can stand the preliminary examination, entering upon the study of medicine at fifteen years of age, and putting in the first three years on the primary branches and two years on the higher branches, and the last year in the hospital in practical work or rather clinical work; however, there was some difference of opinion expressed in the meeting.

The Association was very profitably entertained by services, on Tuesday, in the Episcopal church; sermon by the Archbishop of Canterbury.

On the 30th the sections met in Mason college at ten o'clock, and among the interesting and instructive papers, was one by Dr. W. S. Plyfair on Tediou Labor; one by Dr. Timothy Holmes on Ligation of Large Vessels; one by Mr. Lawson Tait on Operations in so-called Tubercular Peritonitis. The first paper referred to brought out considerable discussion, and the remedies recommended were opium, chloral and quinine. Strychnia, gelsemium and ergot were mentioned but were not strongly advocated and especially the latter (ergot), which was rather condemned than recommended. The mechanical treatment was forceps, and pressure over the fundus of the uterus, and pushing up of the anterior lip over the head. There was also some difference of opinion whether opium hastens or quiets the pains. The paper on ligation of the large vessels was discussed by Marcy, of Boston and Parke, of Chicago, and several of the English surgeons.

The paper by Mr. Lawson Tait was not discussed. He showed two of the patients whom he had operated on for so-called turbercular peretonitis, that had been well for five and seven years respectively. Also a very interesting paper by Mr. Jordan Loyd, showing that the pelvis of the kidney so-called was not funnel shape as he demonstrated by cast of the so-called pelvis with paraffine wax.

The section adjourned at 12:30, and the general meeting convened at 3, to hear the address on general medicine by Mr. B. Walter Foster which was a very able and interesting address, comparing the medicine of thirty-five and forty years ago with the progress and standing of to-day.

The mayor's reception at the Council House was very fully attended; about four thousand were present.

After the section meeting in the morning, at which time some very good papers were read, the meeting adjourned to three o'clock to hear the reading of the address on surgery, by Mr. Lawson Tait, which was very entertaining and amusing, but at the same time some very interesting facts were brought out. The theme of his address was more practical education of the student and less cramming, as he expressed it. He thinks the student should go to the work-shop and learn to use the saw and hammers as well as the jack-plane, so he would not only be able to apply a splint, but to make it. He says all the minute anatomy and theoretical teachings should be abandoned and replaced by practical teaching. He gives Sir Spencer Wells the credit of being the father of abdominal surgery. He claims for J. J. Simpson the discovery of anæsthesia in 1847. He did not so much as mention Crawford W. Long, who is entitled to the honor, and preceded Simpson by several years, not only in chloroform, but ether. Neither was Wells, Morton or Jackson's name referred to as having preceded Simpson also.

I do think that Georgia should come to the front and show up Long's claim, because it is beyond doubt that he was the first to use an anæsthetic of any kind in a surgical operation. Mr. Tait did not refer to any surgeon in our country except McDowell of Kentucky.

The next thing was conferring the honor of the gold medal of the British Medical Association on Dr. Parke, the surgeon of H. M. Stanley in the African expedition, which was received with very great applause.

The membership of the British Medical Association is about thirteen (13,000) thousand, and it certainly is *a very strong body.*

I did not state that Mr. T. Holmes was appointed to make the motion of resolution of thanks for Mr. Lawson Tait's paper, which was seconded by a gentleman from Detroit, Mich., who claims for America the plans of Leach which was advocated by Mr. Tait.

As I will not be at the meeting to-morrow I will close this letter. I will sail on Saturday for America. Hoping this may be of some interest to your readers,

I am respectfully,

L. G. HARDMAN.

